# Liposarcoma: State of the Art—A Scoping Review

**DOI:** 10.3390/medsci14020275

**Published:** 2026-05-28

**Authors:** Bogdan Marian Caraban, Mariana Aschie, Cristian Ionut Orasanu, Raluca Ioana Voda, Anamaria Sincu, Sorin Vamesu, Ionut Bulbuc, Nicolae Ciufu, Mariana Deacu

**Affiliations:** 1 Clinical Department of Plastic Surgery, Microsurgery–Reconstructive, “Sf. Apostol Andrei” Emergency County Hospital, 900591 Constanta, Romania; 2 Faculty of Medicine, “Ovidius” University of Constanta, 900470 Constanta, Romaniaraluca.v1694@yahoo.ro (R.I.V.); anamariasincu5@gmail.com (A.S.); deacu_mariana@yahoo.com (M.D.); 3 Clinical Service of Pathology, Departments of Pathology, “Sf. Apostol Andrei” Emergency County Hospital, 900591 Constanta, Romania; 4 Academy of Medical Sciences of Romania, 030171 Bucharest, Romania; 5 The Romanian Academy of Scientists, 030167 Bucharest, Romania; 6 Center for Research and Development of the Morphological and Genetic Studies of Malignant Pathology (CEDMOG), “Ovidius” University of Constanta, 900591 Constanta, Romania; 7 Department of Pathology, Ovidius Clinical Hospital, 905900 Constanta, Romania; 8 Department of Surgery, Ovidius Clinical Hospital, 905900 Constanta, Romania

**Keywords:** atypical lipomatous tumor, dedifferentiated liposarcoma, myxoid liposarcoma, myxoid pleomorphic liposarcoma, pleomorphic liposarcoma, well-differentiated liposarcoma

## Abstract

**Background/Objectives:** The most common soft tissue sarcomas in adults are liposarcomas, a heterogeneous group of malignant tumors. A structured literature search was conducted to better understand the clinical-imaging aspects and molecular behavior underlying the therapeutic approach. **Methods:** A scoping review was performed according to the PRISMA-ScR guidelines. Searches were conducted in MEDLINE, Web of Science Core Collection, and Google Scholar for the period of 1 January 2016–27 March 2026. Studies that addressed liposarcomas and their subtypes were included. Data were extracted descriptively and synthesized narratively. **Results:** We identified 113 studies that met the inclusion and exclusion criteria. Most studies have focused on a subtype of liposarcomas or on aspects such as clinical, imaging, histopathological, molecular, therapeutic, or prognostic features. The collected data identify, in each case, the need to develop new techniques useful for their identification and deeper molecular analysis. These needs arise from the need to improve treatment and to provide better prognosis, especially in rare or high-grade subtypes. **Conclusions:** The heterogeneity of these tumors requires the provision of a diagnosis that takes into account all possible instruments: clinical, imaging, and histo-molecular. Therefore, further research and translational medicine are needed to discover new pathogenic mechanisms in order to develop individualized therapies that increase quality of life.

## 1. Introduction

According to the World Health Organization, lipomatous soft tissue tumors include three major lesion categories: benign, intermediate or locally aggressive, and malignant [1]. Liposarcoma is the most common sarcoma diagnosed in adults, representing up to 12.8% of all malignant soft tissue tumors. The subtypes of this tumor are atypical lipomatous tumor/well-differentiated liposarcoma, dedifferentiated liposarcoma, myxoid liposarcoma, pleomorphic liposarcoma, and myxoid pleomorphic liposarcoma [1,2].

Atypical lipomatous tumor/well-differentiated liposarcoma (ALT/WDL) is defined as a malignant mesenchymal tumor composed of mature adipocytes, atypical stromal cells, and rare lipoblasts. It has a prevalence of 40–45% and represents 31–33% of all liposarcomas, with a predominance in middle-aged adults, with both sexes being equally affected [3,4,5,6]. Dedifferentiated liposarcoma (DL) is a well-differentiated liposarcoma with a transition to a non-lipogenic sarcoma, usually presenting a high-grade morphology, reminiscent of undifferentiated pleomorphic sarcoma or myxofibrosarcoma. It affects the same patient population as ALT/WDL, with a male:female ratio of 2.1 [7,8]. Approximately 90% of cases occur de novo, and 10% are relapses [6]. Myxoid liposarcoma (ML) is defined as a malignant tumor composed of uniform, round–ovoid cells, associated with a variable number of lipoblasts, and contained in a myxoid stroma. It represents approximately 20–30% of liposarcomas and 5% of soft tissue sarcomas in adults [9,10,11]. It is the most common subtype of liposarcoma in children and adolescents. In adults, it is most commonly diagnosed between 30 and 50 years of age, with a slight predominance for males [10,12]. Pleomorphic liposarcoma (PL) is a high-grade sarcoma, composed of a variable number of pleomorphic lipoblasts without identifying a well-differentiated liposarcoma component or other lines of differentiation. It is a rare subtype of liposarcoma, representing 5–10% of all liposarcomas [1,13]. The peak incidence of these cases is in the seventh decade of life, with a slightly higher predilection for men than women, with a male:female ratio of 1.5 [2,13,14]. Myxoid pleomorphic liposarcoma (MPL) is represented by an association of the characteristics of myxoid and pleomorphic liposarcoma, in the absence of gene fusions and amplifications identifiable in myxoid, dedifferentiated, and well-differentiated liposarcoma. It occurs predominantly in children and young adults. The age of the patients in the vast majority of published cases is <30 years, with a predominance of females [15,16].

The present study aims to provide an in-depth analysis of soft tissue liposarcoma, highlighting the main pathogenic characteristics underlying the therapeutic principles applicable to this pathology. Also, by detailing the existing therapies, we aim to highlight the major importance and urgent need to develop new therapeutic modalities and targets that would prolong disease-free survival (DFS), with a reduction in treatment-induced side effects/adverse reactions, with the ultimate goal of better quality and prolongation of survival.

## 2. Materials and Methods

### 2.1. Study Design

A scoping review was conducted to map the existing literature on liposarcomas, following the Arksey and O’Malley methodological framework, as well as the updated recommendations of the Joanna Briggs Institute (JBI) [17,18]. The reporting of the process follows the PRISMA-ScR (Preferred Reporting Items for Systematic reviews and Meta-Analyses extension for Scoping Reviews) guidelines [19]. This design was chosen to generate an overview of epidemiological, clinical, histopathological, and therapeutic data, facilitating the identification of gaps in the current literature and the need for the development of new clinical management tools. Although the protocol of this scoping review was not prospectively registered in a public database (such as PROSPERO or the Open Science Framework), the methodological steps were rigorously planned and followed internally to ensure transparency and reproducibility of the selection process.

### 2.2. Research Question (PCC Strategy)

To guide the review process, the PCC framework (Population, Concept, Context) was used, formulating the following central question: “Is current knowledge of liposarcomas sufficient for clinical management, or are new tools needed to identify and treat them?”

Population: Patients with liposarcomas (all histopathological subtypes).

Concept: Integrated analysis of epidemiological, molecular, imaging, and therapeutic data.

Context: Clinical and basic research worldwide.

### 2.3. Search Strategy and Information Sources

A comprehensive search was conducted in three major databases: MEDLINE (PubMed), Web of Science Core Collection, and Google Scholar. The search targeted publications from the last 10 years (1 January 2016–27 March 2026), using terms from the MeSH controlled vocabulary and keywords combined through Boolean operators (OR/AND): “liposarcoma”, “atypical lipomatous tumor”, “well-differentiated liposarcoma”, “dedifferentiated liposarcoma”, “myxoid liposarcoma”, “pleomorphic liposarcoma”, and “myxoid pleomorphic liposarcoma”.

### 2.4. Eligibility Criteria

Inclusion Criteria: Studies evaluating the epidemiology, clinical-imaging aspects, pathogenic processes, molecular correlations, therapeutic options, and prognosis of patients with liposarcoma. Primary studies, systematic reviews, and meta-analyses published exclusively in English were included.

Exclusion Criteria: Multiple publications of the same study, conference abstracts, unpublished data, doctoral theses without full text available, and studies on non-human models.

### 2.5. Source Selection and Data Management

All identified references were exported to Microsoft Excel Version 2508 (Microsoft Corporation, Redmond, Washington, DC, USA), where duplicates were removed and the screening process was managed. The selection followed two stages:

Screening of titles and abstracts: Performed independently by three reviewers (A.S., I.B., and N.C.).

Full-text review: The selected articles were reviewed in detail by R.I.V., S.V., and M.D.

Any discrepancies between reviewers at any of the screening stages were resolved by discussion and consensus, and in cases where agreement could not be reached, a fourth senior reviewer (M.A.) was consulted for the final decision.

### 2.6. Data Extraction and Synthesis (Data Charting)

Data were systematically extracted using a data charting form in Microsoft Excel Version 2508 (Microsoft Corporation, Redmond, Washington, DC, USA), targeting: author, year, type of study, subtype of liposarcoma studied, diagnostic methods, and therapeutic outcomes. To ensure methodological rigor, CASP checklists were used to assess the quality of evidence under the supervision of S.V. and M.D. Although the assessment of methodological quality is optional in a scoping review, we chose this approach to provide a transparent picture of the degree of certainty of the available evidence. The quality of the studies was not an exclusion criterion, this decision allowed the inclusion of all relevant studies to highlight methodological limitations in the current literature on liposarcoma.

### 2.7. Analysis and Presentation of Results

Data synthesis was performed through a thematic analysis, with the results being accompanied by summary tables and diagrams, as appropriate, to facilitate the visualization of research trends. The process aimed not only at aggregating positive results but also at explicitly identifying knowledge gaps essential to answering the question of whether new tools are needed in the management of liposarcomas.

## 3. Results

### 3.1. Selection of Sources of Evidence

**Figure 1:**
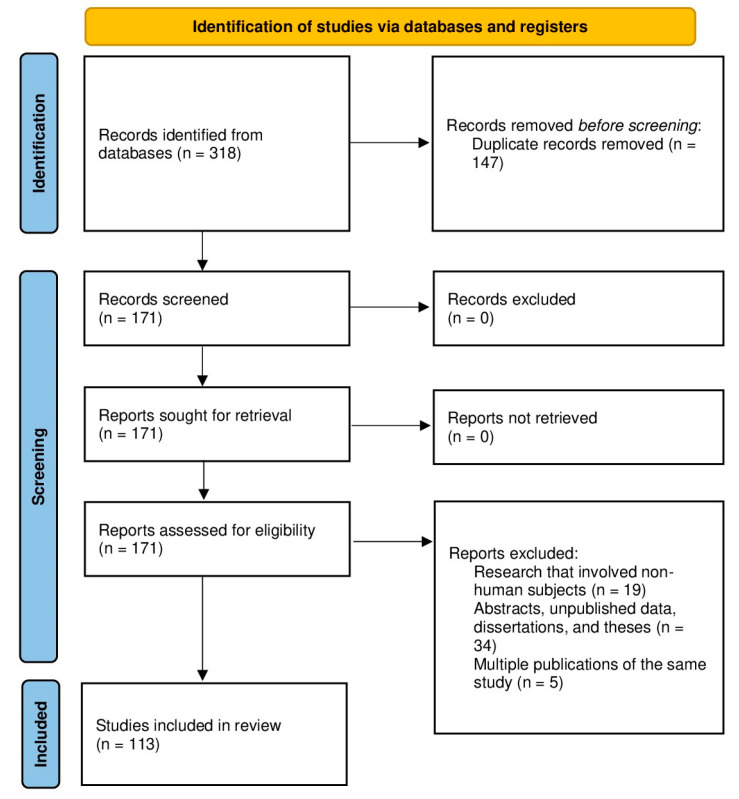
PRISMA diagram showing the process of identifying, screening, and including studies for this analysis of liposarcomas. From the three databases studied, 171 articles entered the screening process, of which 113 met the study eligibility criteria.

The literature search in MEDLINE (PubMed), Web of Science, and Google Scholar initially generated a total of 318 records. After removing duplicates, 171 records were screened based on the title and abstract. Finally, a total of 113 studies met all inclusion criteria and were included in this scoping analysis. The complete selection process and reasons for exclusion at the full-text stage are detailed in the PRISMA-ScR flow chart (Figure 1).

### 3.2. Characteristics of Included Studies

A descriptive analysis of the 113 included studies revealed a diverse distribution of study designs and chronological focus. Most studies were from 2021 and 2024 (16.81% and 15.04%, respectively). Most studies were conducted in North America (37.17%), followed by Asia (33.63%) and Europe (23.89%). In terms of study design, the final selection comprised (42.48%) original research and retrospective cohort studies, 44.25% reviews, and 13.27% case series and case reports focusing on rare subtypes.

In terms of the clinical entities investigated, 32.74% of the studies addressed ALT/WDL and DL as a continuous clinical spectrum. ML was the main topic of 19.47% of the publications and thoroughly described the properties of the myxoid component. In total, 18.58% of the evidence sources studied PL and MPL, with an emphasis on their rarity and high recurrence rates. Additionally, 29.21% of the research investigated more than three entities.

### 3.3. Methodological Quality of Included Sources

The assessment of methodological quality using the CASP checklists revealed an overall moderate-to-high level of evidence in the selected literature, although variations were observed depending on the study design.

The retrospective cohort studies and original articles reviewed had high methodological rigor regarding well-defined research topics and confounder identification; nonetheless, their principal limitations were the retrospective form of data collection and diverse follow-up lengths. The evaluations included in the final analysis met most of the CASP criteria, with strong search strategies and clear inclusion criteria which gave a high degree of certainty. Case series and case reports on rare subtypes (e.g., PL and MPL) have lower CASP scores due to small sample sizes and lack of comparative control groups. These sources had lower scores but were retained in the study to fully address the knowledge gaps and therapeutic obstacles inherent in these unusual clinical entities.

### 3.4. Thematic Synthesis of Evidence

The clinical and imaging features show that liposarcomas are mostly slow-growing tumors of the retroperitoneum and extremities, with late clinical presentation due to the compression of organs. Computed tomography and magnetic resonance imaging are routinely included in the first evaluation as crucial tools. The research highlights the importance of these modalities in the characterization of the adipose component and in the identification of the main indications of malignancy, including thick and irregular intratumoral septa and solid, non-lipomatous nodular regions.

This synthesis confirms, from a histo-molecular and genetic point of view, the subdivision of liposarcomas into five different histopathological and immunohistochemical subtypes, characterized by specific genetic alterations. Accordingly, while ALT/WDL and DL are strictly determined by somatic amplification of the *MDM2* and *CDK4* genes on chromosome 12q13-15, which is considered the gold standard in diagnosis, ML is defined in the literature by the presence of the specific *FUS-DDIT3* chromosomal translocation, unlike the rare variants of PL and MPL, which do not show any particular diagnostic translocation but are strongly linked to complex karyotypes and mutations in the tumor suppressor genes *TP53* and *RB*.

From the point of view of therapeutic management and prognostic outcomes, the universal gold standard for local tumor control throughout the specialized literature remains surgical resection with negative microscopic margins, while adjuvant radiotherapy is used to minimize the risk of recurrence in high-grade forms of malignancy or in challenging anatomical locations, such as the retroperitoneum, and systemic chemotherapy is strictly reserved for metastatic disease or specific chemosensitive subtypes, such as myxoid variants. Therefore, the prognosis is strongly dictated by the molecular subtype, such that ALT/WDL presents excellent long-term survival rates but an increased risk of local recurrence, unlike DL and PL, which demonstrate extremely aggressive clinical behavior, characterized by a high incidence of early metastases and significantly lower survival rates.

## 4. Discussion

### 4.1. Clinical and Imaging Aspects

**Figure 2:**
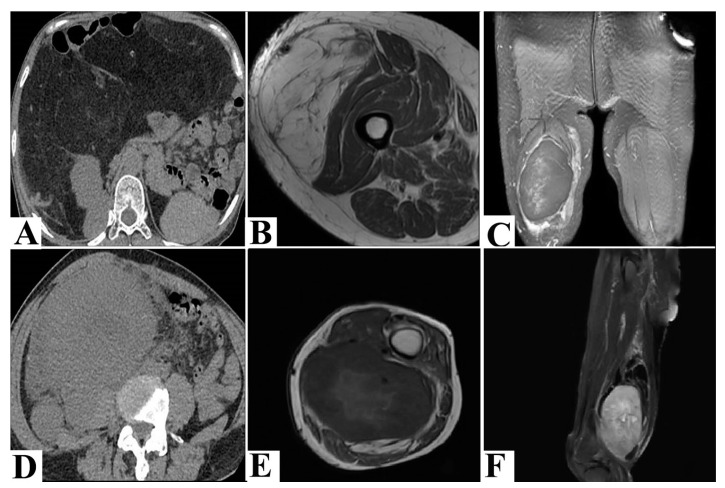
(**A**) Computed tomography image shows a well-differentiated liposarcoma located in the upper abdomen, with well-defined borders, fat-like content, and some septa over 2 mm pushing the surrounding tissues. Adapted from Yang, T. et al. (2025) [26] and licensed under CC BY 4.0. (**B**) Well-differentiated liposarcoma located at the vastus lateralis level in axial and T1 sequences with hyperintensity of the lesion and hypointensity of the septa. Adapted from Natella, R. et al. (2023) [22] and licensed under CC BY 4.0. (**C**) Well-differentiated liposarcoma on T2-weighted fat-suppressed image reveals a well-defined, low-signal adipose tissue mass with thickened septa in the right thigh. Adapted from Zhang T. et al. (2024) [25] and licensed under CC BY NC 4.0. (**D**) A dedifferentiated liposarcoma located in the right lower abdomen, with margins imprecisely delimited from the surrounding tissues, and solid content invading the right psoas major muscle. Adapted from Yang, T. et al. (2025) [26] and licensed under CC BY 4.0. (**E**) Dedifferentiated liposarcoma on T1-weighted images presents as an indistinct adipose tissue mass in the left popliteal fossa with low signal non-fatty areas and heterogeneous areas. Adapted from Zhang T. et al. (2024) [25] and licensed under CC BY NC 4.0. (**F**) Liposarcoma on T2-weighted images with fat suppression shows low-signal suppressed adipose areas and high-signal non-fatty areas, as well as thick septations in the adipose areas. Adapted from Zhang T. et al. (2024) [25] and licensed under CC BY NC 4.0.

The clinical manifestations and imaging aspects of liposarcomas are closely related to their location and, to some extent, to the histopathological subtype (Table 1). ALT/WDL is located predominantly at the extremities (under the deep fascia) and retroperitoneal level and rarely at the mediastinal or paratesticular levels. It presents as a painless tumor mass, with an indolent evolution, of variable size. Large masses are those that cause symptoms with mass effect on adjacent structures, especially the neurovascular bundles [20,21,22]. In superficially located cases, an ultrasound examination can be performed. This highlights a heterogeneous, multilobulated mass with well-demarcated edges. Computed tomography (CT) of these lesions indicates an adipose mass with non-lipomatous components such as thickened septa (over 2 mm) or nodularities (usually under 2 cm) with possible calcifications (Figure 2A). Magnetic resonance imaging (MRI) is an examination that identifies signs of malignancy. These consist of non-adipose nodular areas or adipose areas with different densities, thickened septa over 2 mm or irregular, rapid increase in size from one examination to the next, calcifications, and dimensions over 5 cm in deep locations or over 10 cm in superficial locations (Figure 2B,C). Also, mediastinal, intra-abdominal, pelvic (spermatic cord), or retroperitoneal locations are considered areas of risk for malignancy [22,23].

DL can be located anywhere but has a predilection for the retroperitoneal region, where it tends to extend into the abdominal cavity and metastasize distantly. Other common locations are the spermatic cord, the head and neck region, or the extremities. In the case of the extremities, it is located proximally in the depth of the soft tissue. Patients present with a large painless tumor mass that grows over time. More rarely, it can be an incidental finding. The effect of its size is reflected on the surrounding organs and can cause abdominal pain, abdominal distension, intestinal obstruction, or urinary obstruction [24,25]. In superficial locations, sonography identifies a hyperechoic mass with hypervascularization. CT can identify non-characteristic aspects, similar to those found in the case of WDL, such as slightly irregular edges, mixed densities, and nodules. However, aspects such as heterogeneous densities caused by non-adipose components associated with irregular edges tip the balance in favor of DL (Figure 2D) [26,27]. To increase the sensitivity of the differential diagnosis between the two entities, studies have shown that positron emission tomography (PET-CT) with a cut-off of four standardized uptake values is an essential tool [28]. MRI identifies fatty masses with thick septa, intense heterogeneous enhancement, necrosis, and edema (Figure 2E,F) [29,30].

**Figure 3:**
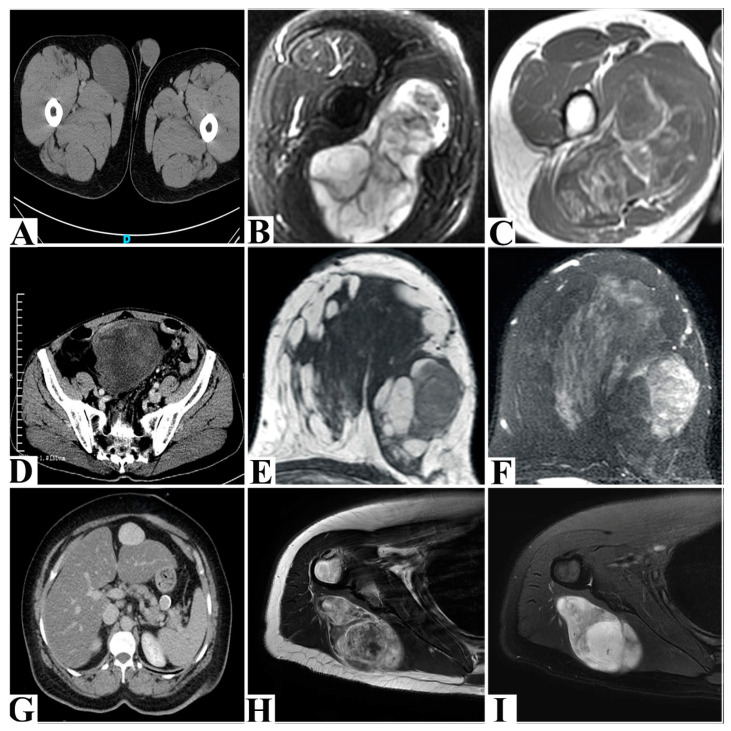
(**A**) Computed tomography shows a myxoid liposarcoma in the medial part of the subcutaneous soft tissues of the right thigh, measuring 7.3 × 5.6 cm. Adapted from Correa, N. et al. (2026) [33], licensed under CC BY 4.0. (**B**) Myxoid liposarcoma in axial T1 sequence reveals hypointensity of the lesion. Adapted from Natella, R. et al. (2023) [22] and licensed under CC BY 4.0. (**C**) Myxoid liposarcoma in axial T2 sequence, with fat suppression, shows hyperintensity of the lesion. Adapted from Natella, R. et al. (2023) [22] and licensed under CC BY 4.0. (**D**) Contrast-enhanced computed tomography of a pleomorphic liposarcoma located in the lower abdomen with a heterogeneous appearance and clear boundaries. Adapted from Wang, L. et al. (2018) [37] and licensed under CC BY 4.0. (**E**) Pleomorphic liposarcoma in axial T1-weighted image reveals heterogeneous areas and areas of hyposignals. Adapted from Jo, S.J. et al. (2020) [40] and licensed under CC BY 4.0. (**F**) Pleomorphic liposarcoma in axial T2-weighted image shows heterogeneous areas and areas of hypersignal. Adapted from Jo, S.J. et al. (2020) [40] and licensed under CC BY 4.0. (**G**) Contrast-enhanced computed tomography revealed a myxoid pleomorphic liposarcoma as a well-defined mass with progressive contrast, located anterior to the left hepatic lobe. Adapted from AlObaid, B. et al. (2022) [44] and licensed under CC BY 4.0. (**H**) Myxoid pleomorphic liposarcoma examined by T1-weighted magnetic resonance imaging showing a heterogeneous soft tissue mass with a myxoid component in the teres minor muscle. Adapted from Choi, J.H. et al. [49] and licensed under CC BY 4.0. (**I**) Myxoid pleomorphic liposarcoma was examined by T2-weighted fat-suppressed magnetic resonance imaging, showing a heterogeneous soft tissue mass with a myxoid component in the teres minor muscle. Adapted from Choi, J.H. et al. [49] and licensed under CC BY 4.0.

ML is a slow-growing malignant tumor located deep in the lower extremities. Less commonly, it can be located in the neck, intrathoracic, intra-abdominal, retroperitoneal, or upper extremities. Symptoms are most often absent. Some patients complain of pain at the lesion level. Due to these insidious manifestations, the tumors grow in size, and the average diameter at the time of diagnosis reaches 12 cm [31]. In superficial areas, on ultrasound, the lesions are well vascularized and heterogeneous, with large areas of adipose appearance with foci of low echogenicity [32]. CT shows a well-defined lobulated mass with soft tissue attenuation (Figure 3A). In contrast, DL presents a lower apparent diffusion coefficient (ADC) value and higher unenhanced CT attenuation. MRI indicates an encapsulated mass with hypointensity in T1 and marked hyperintensity in T2 with heterogeneous or increased enhancement (Figure 3B,C). In high-grade cases, a heterogeneous signal is observed in attenuated T1 and T2 images caused by non-adipose and non-myxoid areas mixed with adipose and myxoid areas. Non-adipose and non-myxoid areas show intermediate signals in T2 with variable enhancement [33,34,35,36]. A special aspect is represented by the identification of bone metastases, which in almost half of the cases cannot be identified by CT, PET-CT, or bone scintigraphy [32].

PL is predominantly located in the extremities, especially the proximal and lower extremities. Other locations may include the abdominal wall, retroperitoneum, pelvic cavity, spermatic cord, mediastinum, or head and neck region [37,38]. Clinical manifestations are late and are generated by the increase in size and compression of adjacent structures [39,40]. From an imaging point of view, they are difficult to diagnose due to the absence of adipose tissue. The general characteristics concern a large, relatively well-demarcated, multinodular mass with heterogeneity due to hemorrhage and necrosis (Figure 3D) [41]. In superficial locations, sonography reveals a gyrus-like architecture with hyper- and hypoechoic areas. MRI reveals a heterogeneous mass with areas of hypointense signals, like skeletal muscle in attenuated T1, and hyperintensity, like that of adipose tissue in attenuated T2 (Figure 3E,F) [27].

MPL presents as a large tumor mass (median 12.5 cm) with deep localization. It is most frequently encountered at the mediastinal level and very rarely in the head and neck region, abdomen, perineum, and orbit. The symptomatology is given by its compressive effect depending on the localization [42,43]. Ultrasonography shows a hypoechoic lesion with internal vascularization [44,45]. CT observes a large hypodense mass with heterogeneous enhancement (Figure 3G). MRI shows a heterogeneous mass with adipose and myxoid components (Figure 3H,I) [46,47,48,49].

**Table 1:** The main imaging aspects of liposarcomas.

Tumor	Frequent Location	Sonographic Characterization	CT Characterization	MRI Characterization
Atypical lipomatous tumor/well-differentiated liposarcoma [20,21,22,23,27]	Extremities/retroperitoneal	Heterogeneous mass, iso- with hyperechogenicity.	Multilobulated appearance, with well-defined edges. Adipose mass with non-adipose components, thickened septa over 2 mm, or nodularities.	Adipose areas with different densities, thickened septa over 2 mm or irregular, and dimensions over 5 cm in deep locations or over 10 cm in superficial locations.
Dedifferentiated liposarcoma [24,25,26,27,28,29,30]	Retroperitoneal	Hyperechoic mass with hypervascularization.	Slightly irregular margins. Mixed densities (heterogeneous areas—non-adipose components) and nodules.	Fatty mass with thick septa, intense heterogeneous enhancement, necrosis, and edema.
Myxoid liposarcoma [27,31,32,33,34,35,36]	Lower extremities	Mass with heterogeneous areas with foci of low echogenicity, hypervascularization.	Well-defined lobulated mass with soft tissue attenuation.	Low grade: Encapsulated mass with hypointensity on T1 and marked hyperintensity on T2 with heterogeneous or increased enhancement. High grade: Heterogeneous signal on T1- and T2-weighted images. Non-adipose and non-myxoid areas show intermediate signal on T2 with variable enhancement.
Pleomorphic liposarcoma [27,37,38,39,40,41]	Proximal and lower extremities	Mass with gyrus-like architecture with hyper- and hypoechoic areas.	Large, relatively well-demarcated, multinodular mass with heterogeneity due to hemorrhage and necrosis.	Heterogeneous mass with areas of hypointense signal on T1-weighted and hyperintense on T2-weighted images.
Myxoid pleomorphic liposarcoma [42,43,44,45,46,47,48,49]	Mediastinal	Hypoechoic mass with internal vascularity.	Large hypodense mass with heterogeneous enhancement.	Heterogeneous mass with adipose and myxoid components.

### 4.2. Histo-Molecular Aspects

The etiopathogenesis of liposarcomas involves complex genetic alterations (Table 2). In over 90% of cases, distinct chromosomal abnormalities are observed. In rare cases, familial hereditary syndromes with a predisposition to cancer are present, such as Li–Fraumeni syndrome (*TP53* mutations), neurofibromatosis type 1, or retinoblastoma (*RB1* germline mutations) [50].

**Figure 4:**
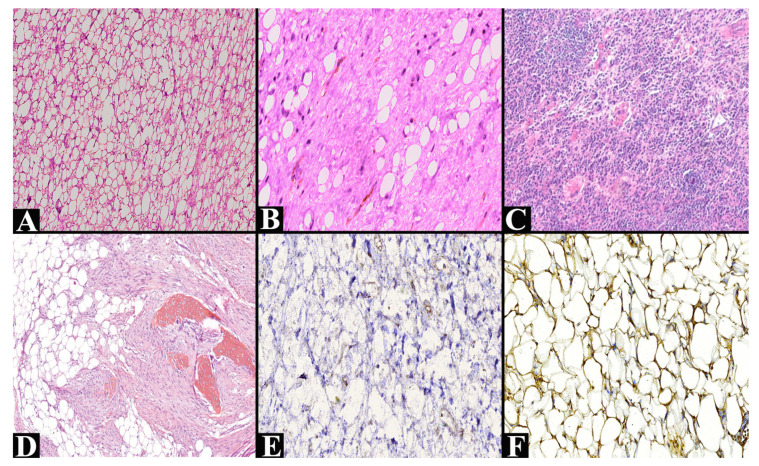
(**A**) Well-differentiated liposarcoma, lipoma-like subtype that presents rare, atypical cells among adipocytes of various sizes (hematoxylin–eosin staining, ×100, original image from the Clinical Anatomy-Pathology Service, Constanta). (**B**) Well-differentiated liposarcoma, sclerosing subtype, reveals dense collagenous fibrous tissue among adipocytes of varying sizes, minimal nuclear atypia (hematoxylin–eosin staining, ×200, original image from the Clinical Anatomy-Pathology Service, Constanta). (**C**) Well-differentiated liposarcoma of inflammatory subtype presents atypical stromal cells in an abundant chronic inflammatory infiltrate (hematoxylin–eosin stain, ×100). Adapted from Choi, J.H. et al. (2020) [53], licensed under CC BY 4.0. (**D**) Clusters of adipocyte cells arranged in a stromal proliferation composed of spindle cells, as well as a low-grade osteosarcoma component (hematoxylin–eosin stain, ×40). Adapted from Macagno, N. et al. (2017) [59], licensed under CC BY 4.0. (**E**) Positive nuclear reactivity to MDM2 in well-differentiated liposarcoma (×100, original image from the Clinical Anatomy-Pathology Service, Constanta). (**F**) Positive reactivity to S100 in well-differentiated liposarcoma (×100, original image from the Clinical Anatomy-Pathology Service, Constanta).

ALT/WDL shows amplification for murine double minute 2 (*MDM2*) and cyclin-dependent kinase 4 (*CDK4*) located at chromosome 12q13-15. Damage at this level will lead to the presence of supernumerary rings and rod chromosomes, a characteristic feature. *CDK4* causes hyperphosphorylation of the retinoblastoma protein, which does not suppress the E2F transcription factor, leading to unregulated cell proliferation. Amplification of *MDM2* inhibits p53 and leads to the degradation of this protein, resulting in low levels of it. Thus, its tumor-suppressive activity is neutralized, promoting uncontrolled growth. Other pathogenic mechanisms involved are represented by the amplification of high-mobility group protein 2a (*HMG2A*), YEATS domain-containing 4 (*YEATS4*), and carboxypeptidase M (*CPM*). These are involved in the dedifferentiation of liposarcomas but also in the suppression of the *TP53* gene, as well as in the cleavage of growth factor enzymes [3,51,52]. From a histopathological point of view, three subtypes can be observed (Figure 4A–C). The adipocytic or lipoma-like subtype presents mature adipocytes with variations in size, accompanied by nuclear hyperchromasia. Adipocyte and stromal cell atypia are identified, as well as lipoblasts. The sclerosing subtype presents bizarre, diffusely arranged stromal cells with nuclear hyperchromasia and extensive collagenous stroma. The inflammatory subtype presents an inflammatory infiltrate with intense chronic cells accompanied by atypical, bizarre, diffusely arranged multinucleated stromal cells [53]. The classic diagnostic immunohistochemical panel should include positive immunoreactivity for p16, MDM2, CDK4, HMGA2, and S100 (Figure 4E,F). Last but not least, MDM2 reactivity must be reconfirmed by FISH [54,55].

This tumor may present heterologous elements, making diagnosis very difficult. The two situations encountered so far identify a smooth muscle and a bone component. Lipoleiomyosarcoma presents a multifocal and gradual transition to a malignant smooth muscle tumor originating from the great vessels. ALT/WDL associated with a low-grade osteosarcomatous component is rare and presents a malignant bone component similar to either a primary parosteal osteosarcoma or a low-grade central osteosarcoma (Figure 4D). Thus, bone trabeculae of variable sizes without osteoblastic borders are noted, which are confused with a fibroblastic proliferation [56,57,58,59].

The main entities with which the differential diagnosis is made are lipoma, spindle cell lipoma, pleomorphic lipomatous tumor, and dedifferentiated liposarcoma. Lipoma has a negative expression for MDM2. Spindle cell lipoma and pleomorphic lipomatous tumors have positive expression for CD34 and negative expression for Rb1 and MDM2. Dedifferentiated liposarcoma has a similar appearance to malignant fibrous histiocytoma without adipose differentiation on at least one low-power field and shows distinct edges of the differentiated component, as well as mitotic activity of more than five mitoses/10 HPF [60].

DL presents more complex pathogenesis than the previous one. In addition to the amplification of the 12q13-15 sequences (presence of rings and supernumerary rod chromosomes), amplifications of chromosomes 1p32 and 6q23 are also identified, leading to a worse prognosis. In addition to the previously mentioned sequences (*MDM2*, *CDK4*, *HMGA2*, and *YEATS*), tetraspanin 31 (*TSPAN31*) and solute carrier family 35 member E3 (*SLC35E3*) are added. Both have a crucial role in the development of dedifferentiated liposarcoma. Co-amplification of 1p32 and 6q23 will lead to the upregulation of Jun proto-oncogene (*JUN*) and mitogen-activated protein kinase kinase kinase 5 (*MAP3K5*). *JUN* has a role in proliferation, transformation, and apoptosis, inhibiting adipocyte differentiation. *MAP3K5* intervenes in the Jun N-terminal kinase (*JNK*) signaling pathway responsible for *JUN* activation, accentuating the blockage of adipocyte differentiation [38,61,62].

**Figure 5:**
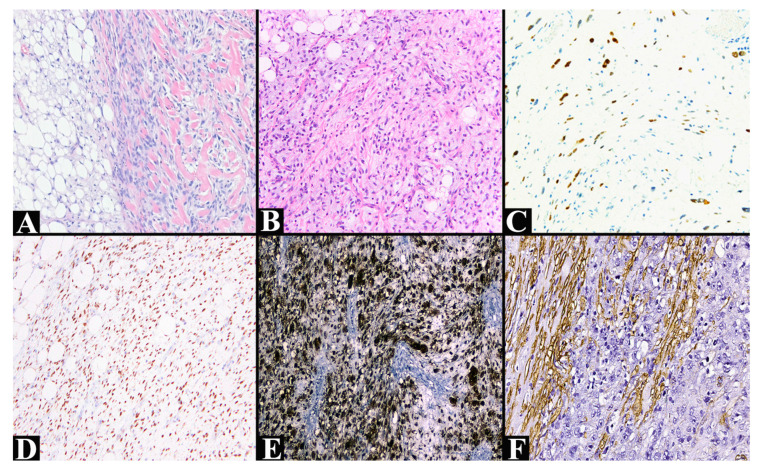
(**A**) Dedifferentiated liposarcoma shows an abrupt transition between the well-differentiated component and the high-grade non-lipogenic component (hematoxylin–eosin staining, ×100). Adapted from Choi, J.H. et al. (2020) [53], licensed under CC BY 4.0. (**B**) Dedifferentiated liposarcoma with transition zone between well-differentiated and non-lipogenic components of neoplastic spindle cells (hematoxylin–eosin staining, ×100). Adapted from Anderson W.J. et al. (2021) [69], licensed under CC BY 4.0. (**C**) The MDM2 reaction is present in the dedifferentiated area (×100). Nishio, J. et al. (2021) [61], licensed under CC BY 4.0. (**D**) CDK4 is present in the nucleus in dedifferentiated liposarcoma (×100). Adapted from Anderson W.J. et al. (2021) [69], licensed under CC BY 4.0. (**E**) Positive reaction to p16 in dedifferentiated liposarcoma (×200, original image from the Clinical Anatomy-Pathology Service, Constanta). (**F**) Positive reaction to alpha-smooth muscle actin in the dedifferentiated muscular component of dedifferentiated liposarcoma (×200, original image from the Clinical Anatomy-Pathology Service, Constanta).

By acquiring these amplification chains, approximately 10% of ALT/WDL evolves to DL. Histopathologically, it presents two distinct constituents represented by a WDL component and a high-grade non-lipogenic (dedifferentiated) component (Figure 5A,B). The first component has lobules of adipocytes of varying sizes with collagenous fibrous septa and often lymphoid aggregates. Adipocyte cells are often located in fibrous areas and have hyperchromic nuclei of varying sizes, often with a floret-like arrangement and low mitotic activity. Abruptly or gradually, areas of the dedifferentiated component are identified. The dedifferentiation can be focal or major and frequently resembles an undifferentiated pleomorphic sarcoma or high-grade myxofibrosarcoma. Low-grade tumors present bland fibroblast-like spindle cells, with low nuclear atypia and a low mitotic rate. Heterogeneous differentiations such as myogenic (leiomyosarcoma or rhabdomyosarcoma), chondrosarcoma, or osteosarcoma can be encountered. Particular aspects concern meningothelial-like architectures with metaplastic or neural-like ossifications [61,63,64,65,66,67].

Immunohistochemically (Figure 5C–F), immunopositivity for MDM2, CDK4, and p16 is identified in both compartments, with a stronger immunointensity in the well-differentiated one. The particularities consist of areas of dedifferentiation: rhabdomyosarcoma—desmin and myogenin; leiomyosarcoma—SMA and caldesmon. Also, CD34 and INI1 can be positive in the heterologous component. An important aspect is the immunonegativity for keratin and S100. Due to the dedifferentiated component, great care must be taken in the differential diagnosis of other soft tissue tumors: rhabdomyosarcoma, leiomyosarcoma, clear cell sarcoma (S100-positive), synovial sarcoma (EMA- and cytokeratin-positive), undifferentiated pleomorphic sarcoma (CD68- and SMA-positive), or gastrointestinal stromal tumors (CD117- and DOG1-positive) [54,55,68].

In 95% of cases, ML presents the chromosomal translocation t(12; 16) (q13; p11) responsible for the *FUS-DDIT3* fusion protein. In the other cases, the translocation t(12; 22) (q13; q12) responsible for the production of the *EWSR1-DDIT3* protein is observed. These two proteins represent a diagnostic indicator with a high degree of specificity for this entity. Their expression is directly proportional to the degree of cellular differentiation and the malignancy of the tumor. Activation of the PI3K/Akt pathway leads to the overexpression of growth factor receptors and transformation into high-grade ML. In cases of transformation, increased levels of miR-135b were observed, this being an oncogenic marker of transformation but also an essential element in enhancing growth, invasiveness, and metastasis [70,71,72,73,74].

**Figure 6:**
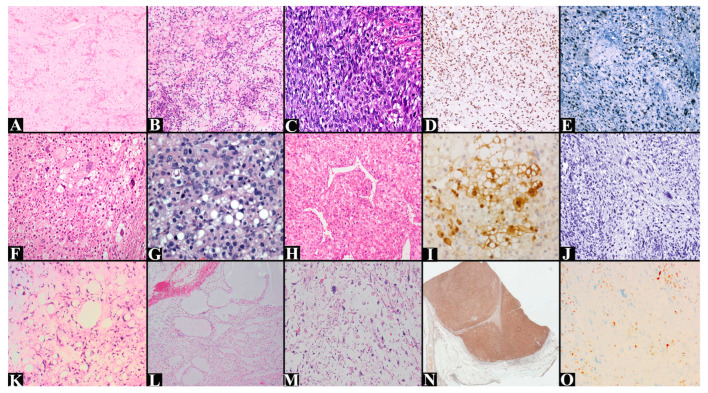
(**A**) Myxoid liposarcoma composed of moderate cellularity and spindle and stellate cells arranged in an abundant myxoid stroma with plexiform vessels (hematoxylin–eosin staining, ×100). Adapted from Correa, N. et al. (2026) [33], licensed under CC BY 4.0. (**B**) Myxoid liposarcoma composed of round-to-ovoid tumor cells, univacuolated lipoblasts, and prominent thin-walled blood vessels. Adapted from Anderson W.J. et al. (2021) [69], licensed under CC BY 4.0. (**C**) High-grade myxoid liposarcoma composed of hypercellularity of primitive round cells. (hematoxylin–eosin staining, ×200, original image from the Clinical Anatomy-Pathology Service, Constanta). (**D**) Myxoid liposarcoma with diffuse nuclear positivity for DDIT3 (×100). Adapted from Anderson W.J. et al. (2021) [69], licensed under CC BY 4.0. (**E**) Nuclear p53-positive myxoid liposarcoma (×100, original image from the Clinical Anatomy-Pathology Service, Constanta). (**F**) Pleomorphic liposarcoma composed of large, atypical, multivacuolized lipoblasts with indented hyperchromatic nuclei, dispersed on a background of atypical spindle cells (hematoxylin–eosin staining, ×100). Adapted from Lee, A.T.J. et al. (2017) [38], licensed under CC BY 4.0. (**G**) Pleomorphic liposarcoma with pleomorphic cells with eosinophilic to vacuolated cytoplasm and indented nuclei (hematoxylin–eosin staining, ×200). Adapted from Jo, S.J. et al. (2020) [40], licensed under CC BY 4.0. (**H**) Epithelioid pleomorphic liposarcoma with hemangiopericytoma-like vessels (hematoxylin–eosin staining, ×200). Adapted from Abe, M. et al. (2023) [85], licensed under CC BY 4.0. (**I**) Pleomorphic liposarcoma with focal positive S100 expression in the lipogenic component (×200). Adapted from Abe, M. et al. (2023) [85], licensed under CC BY 4.0. (**J**) CDK4-negative pleomorphic liposarcoma (×100, original image from the Clinical Anatomy-Pathology Service, Constanta). (**K**) Myxoid pleomorphic liposarcoma that presents areas with the appearance of low-grade myxoid liposarcoma but also areas with lipoblasts with increased atypia and pleomorphism (hematoxylin–eosin staining, ×200). Adapted from Fadaei, S. et al. (2024) [43], licensed under CC BY 4.0. (**L**) Myxoid pleomorphic liposarcoma presents with lymphangioma-like myxoid pools (hematoxylin–eosin staining, ×40). Adapted from Choi, J.H. et al. (2022) [49], licensed under CC BY 4.0. (**M**) Myxoid pleomorphic liposarcoma shows scattered pleomorphic cells and pseudocystic changes (hematoxylin–eosin staining, ×100). Adapted from Choi, J.H. et al. (2022) [49], licensed under CC BY 4.0. (**N**) Myxoid pleomorphic liposarcoma shows cellular immunopositivity for CD34 (×1.25). Adapted from AlObaid, B. et al. (2022) [44], licensed under CC BY 4.0. (**O**) Myxoid pleomorphic liposarcoma, lost immunoreaction for Rb, positive vascular endothelial control (×200). Adapted from Fadaei, S. et al. (2024) [43], licensed under CC BY 4.0.

Histopathologically, low-grade ML presents hypocellularity composed of bland spindle cells located in an abundant myxoid stroma in which thin-walled capillaries of a plexiform, chicken-wire appearance are identified (Figure 6A). High-grade liposarcomas, or round cell liposarcomas, present hypercellular areas exceeding 5% of the lesion (Figure 6B,C). These may mask the capillary network. The cells present scant, eosinophilic cytoplasm with hyperchromic nuclei. Lipoblasts are difficult to identify in these areas [75,76].

Immunohistochemistry (Figure 6D,E) is used more in differential diagnosis than in supporting the diagnosis. The only marker with increased sensitivity is DDIT3, which can also be doubled by FISH examination. Other markers that may be positive are S100 in the lipoblastic or hypercellular component, as well as p53, especially in high-grade variants. Otherwise, MDM2 and CDK4 are negative, unlike DL with myxoid aspects. Other entities with which the differential diagnosis can be made are clear cell sarcoma (HMB45-positive) or gastrointestinal stromal tumor (CD117-positive) [55,69,77,78,79].

Unlike other liposarcomas, PL does not present constant or particular cytogenetic abnormalities. These aspects may be due to the limited studies on the rarity of its molecular substrate. The most common chromosomal abnormalities consist either of losses of chromosomes 1q, 2q, 3p, 4q, 10q, 11q, 12p13, 13q14, 13q21–q, or 13q23–24, or additions of chromosomes 1p, 1q21–q32, 2q, 3p, 3q, 5p12–p15, 5q, 6p21, 7p, or 7q22. One of the most common abnormalities is represented by the deletion of the 13q14.2–q14.3 locus of the *RB* gene; thus, its expression is weakly expressed. In 60% of cases, mutations in the *TP53* gene were identified, a gene rarely mutated in other liposarcomas. In 5% of cases, mutations in the *NF1* gene were observed. In addition to the triad that confers increased aggressiveness and some resistance to chemotherapy, overexpression of genetic markers such as BCL2, PPAR-γ, Survivin, and VEGF was also identified [3,80,81].

From a histopathological point of view, it presents a wide lesional polymorphism with lipoblasts as its central point, which are mandatory for making the diagnosis. Microscopically, it resembles an undifferentiated pleomorphic sarcoma with cells of various sizes and shapes, spindle cells, pleomorphic cells (large, eosinophilic cytoplasm, with bizarre nuclei sometimes with pseudoinclusions), multinucleated giant cells, and lipoblasts (with nuclei identified by lipid vacuoles) (Figure 6F–H). Hypocellular myxoid areas resembling myxofibrosarcoma or epithelioid cells can be found. The latter have abundant eosinophilic cytoplasm and are arranged in a sheet-like arrangement. Other common aspects consist of cytoplasmic or stromal hyaline droplets, tumor necrosis, and infiltrative character [39,82].

Immunohistochemistry (Figure 6I,J) shows p53 overexpression, loss of Rb, and immunopositivity for p16, S100, and CD34, and focal SMA. MDM2 and CDK4 markers are negative, which distinguishes it from WDL and DL tumors. Also, DDIT3 is negative, excluding ML. S100 protein positivity distinguishes it from myxofibrosarcoma. Epithelioid areas, which can sometimes dominate the lesional picture, are positive for AE1/AE3, S100, and Melan A. These must be differentiated from clear cell renal cell carcinoma or adrenal cortical carcinoma [39,83,84,85].

MPL has a hyperploid or hypotriploid karyotype with numerous aberrations. Among the most common are additions of chromosomes 1, 6–8, and 18–21, and deletions of chromosomes 13, 16, and 17. The involvement of chromosomes 13 and 17 is responsible for mutations in the *RB* and *TP53* genes, respectively. In addition, alterations have been identified in the alpha-thalassemia/mental retardation, X-linked (*ATRX*) genes—12%, cysteinyl leukotriene receptor 2 (*CYSLTR2*)—25%, and phosphatase and tensin homolog (*PTEN*)—12%. In a single case, the fusion of cAMP-responsive element-binding protein 5 with telomerase reverse transcriptase (*CREB5::TERT*) and the rearrangement of ETS variant transcription factor 1 with O-fucosylpeptide 3-N-acetylglucosaminyltransferase (*ETV1::LFNG*) were identified [16,46,47].

Microscopically, it shows a pattern of low-grade ML in a proportion of 10–75%, with abundant myxoid matrix with bland ovoid-fusiform cells and blood vessels with a chicken-wire arrangement (Figure 6K–M). This area shows a transition to a hypercellular area composed of high-grade pleomorphic cells with anisonucleosis, nuclear hyperchromasia, and frequent atypical mitoses. The architecture of these areas can be sheet-like or solid. Areas of necrosis, pleomorphic lipoblasts, and multinucleated tumor cells can be identified [42,43,47].

The cellularity is diffusely positive for p16 and CD34 and focally positive for S100. Rb expression is lost, and p53 may have overexpression or null expression (Figure 6N,O). The absence of CDK4 and MDM2 distinguishes it from DL, and the absence of DDIT3 distinguishes it from ML. Differential diagnosis of PL is challenging and is based on the histopathological appearance and the predilection for the pediatric population and young patients [15,16,83,86].

**Table 2:** Main pathogenic mechanisms and histo-molecular aspects.

Tumor	Pathogenesis	Subtypes/Particularities	Immunohistochemistry	Differential Diagnosis
Atypical lipomatous tumor/well-differentiated liposarcoma [3,51,52,53,54,55,56,57,58,59,60]	*MDM2* *CDK4* *HMGA2* *YEATS4* *CPM*	• Subtypes: Lipoma-like Sclerosing Inflammatory • Particularities: Lipoleiomyosarcoma Atypical lipomatous tumor/well-differentiated liposarcoma with low-grade osteosarcomatous component	p16+ MDM2+ CDK4+ HMGA2+ S100+	Lipoma Spindle cell lipoma Pleomorphic lipomatous tumor Dedifferentiated liposarcoma
Dedifferentiated liposarcoma [38,54,55,61,62,63,64,65,66,67,68,69]	*MDM2* *CDK4* *HMGA2* *YEATS4* *CPM* *TSPAN31* *SLC35E3*	• Particularities: Meningothelial-like Neural-like	Dedifferentiated components: P16+ MDM2+ CDK4+ CD34+ INI1+ SMA, caldesmon+/− Desmin, myogenin+/− S100−	Well-differentiated liposarcoma Rhabdomyosarcoma Leiomyosarcoma Clear cell sarcoma Synovial sarcoma Undifferentiated pleomorphic sarcoma Gastrointestinal stromal tumor
Myxoid liposarcoma [55,70,71,72,73,74,75,76,77,78,79]	*FUS-DDIT3* *EWSR1-DDIT3*	• Subtypes: Low-grade High-grade (round cell liposarcoma)	DDIT3+ S100+ P53+ MDM2− CDK4−	Dedifferentiated liposarcoma with myxoid features Clear cell sarcoma Gastrointestinal stromal tumor
Pleomorphic liposarcoma [3,39,80,81,82,83,84,85]	*RB* *TP53* *NF1* BCL2 PPAR-γ Survivin VEGF	• Particularities: Hypocellular myxoid areas Epithelioid cells	P16+ S100+ CD34+ SMA focal Rb− MDM2− CDK4− DDIT3− AE1/AE3, S100, Melan A+ (epithelioid)	Well-differentiated liposarcoma Dedifferentiated liposarcoma Myxoid liposarcoma Myxofibrosarcoma Clear cell renal cell carcinoma Adrenal cortical carcinoma
Myxoid pleomorphic liposarcoma [15,16,42,43,46,47,83,86]	*RB* *TP53* *ATRX* *CYSLTR2* *PTEN* *CREB5::TERT* *ETV1::LFNG*	-	p16+ CD34+ S100 focal p53+/− Rb− MDM2− CDK4− DDIT3−	Dedifferentiated liposarcoma Myxoid liposarcoma Pleomorphic liposarcoma

The most widely used histopathological grading system is that of the Fédération Nationale des Centres de Lutte Contre le Cancer (FNCLCC). It includes tumor differentiation, mitotic activity, and tumor necrosis. Tumor differentiation is scored from one to three for similarity to adult mesenchymal tissue. Thus, one point is assigned to WDL, two points are assigned to ML, and three points are assigned to DL, ML with round cells, and PL. Mitotic activity is measured in 10 HPF. A score of 0–9 mitoses is assigned one point, a score of 10–19 mitoses is assigned two points, and more than 20 mitoses is assigned three points. In the case of tumor necrosis, its absence (0 points) or presence (<50% for one point and >50% for two points) is assessed. The histological grade will sum the score, so a well-differentiated grade (G1) will have 2–3 points, a moderately differentiated grade (G2) will have 4–5 points, and a poorly differentiated grade will have a score of 6–8 points (Table 3) [87].

**Table 3:** The French Federation of Cancer Centers Sarcoma Group grading system.

Parameter	Score	Description
Tumor differentiation	1	Very similar histologically to normal adult mesenchymal tissue (well-differentiated liposarcoma)
	2	Sarcoma of defined histological subtype (myxoid liposarcoma)
	3	Sarcoma of uncertain type, embryonal and undifferentiated sarcomas (dedifferentiated liposarcoma, myxoid liposarcoma with round cells, pleomorphic liposarcoma)
Mitosis	1	0–9 mitoses/10 HPF
	2	10–19 mitoses/10 HPF
	3	>20 mitoses/10 HPF
Tumor necrosis	0	No necrosis
	1	<50% tumor necrosis
	2	>50% tumor necrosis
Final histological grade	1	Total score 2 or 3
	2	Total score 4 or 5
	3	Total score >6

### 4.3. Therapeutic Management

Therapeutic strategies for liposarcomas must take into account certain desiderata such as clinical evolution, location, histopathological type, immunohistochemical examination, and molecular changes. Thus, each treatment applied must be individualized [88]. The gold standard in treatment consists of surgical resection. In high-grade liposarcomas, other therapeutic methods can be associated [6,89]. Regarding radiotherapy, the most radiosensitive liposarcoma is the myxoid, while the others present a moderate radiosensitivity. Also, ML is the most chemosensitive, followed by PL. DL is more frequently chemoinsensitive, and WDL is chemoresistant [90].

Surgical treatment is the method of choice in ALT/LWD. The goal of the intervention is complete, intact resection of the tumor, which includes the contiguous organs involved. There are situations when, intraoperatively, liposarcoma may appear extremely similar to fat, which makes it difficult to differentiate tumoral from non-tumoral areas. In these situations, the extent of resection should be guided by preoperative imaging and the risk of recurrence. Complete excision of any retroperitoneal adipose tissue at risk of harboring the tumor is recommended. Of course, the patient’s quality of life should not be affected after post-surgical recovery. Regarding extensive excision, some studies have observed a much lower recurrence rate than complete excision. However, in some cases, such as tumors located in the extremities, marginal excision may be an acceptable treatment only to preserve the integrity of important anatomical structures (great vessels, nerves, and bone structures), with a higher risk of recurrence. In tumors located in the extremities, abdominal wall, or trunk, adjuvant or neoadjuvant therapies have no role. In any other situation, as well as in cases of dedifferentiation, targeted therapies or neoadjuvant radiotherapy can be applied, as in other liposarcomas [6,89,91].

In DL, optimal surgical excision includes complete resection, ideally involving 1–2 cm of adjacent normal tissue. In retroperitoneal locations, this goal is difficult to achieve, requiring multivisceral resection. Therefore, a complete macroscopic resection will be aimed for. In extremity locations, limb-sparing surgery is the standard. Radical resection involves 1–2 cm margins of adjacent muscle, sparing large blood vessels and nerves. There are situations at this level when the depth of the tumor involves resection of the periosteum. Radiotherapy in retroperitoneal locations is not recommended because studies have shown no increase in recurrence-free survival. Radiotherapy in extremity locations is associated with a lower risk of local recurrence. This approach is applied in large tumors, high-grade ones, or in those where only an incomplete resection can be performed. Preoperative radiotherapy is preferred because it is associated with fewer long-term adverse effects. Treatment with 2 Gy fractionated radiotherapy, which means delivering 2 gray (Gy) of radiation in each treatment session, is used in a total regimen of 50 Gy over 5 weeks. If radiotherapy is considered necessary in retroperitoneal liposarcomas, the same dosage regimen will be applied [92,93,94].

**Figure 7:**
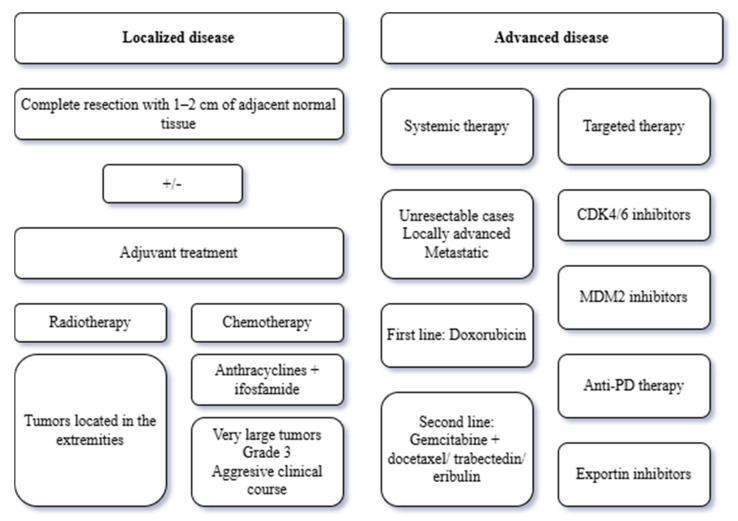
Schematic of therapeutic options for dedifferentiated liposarcoma.

In the absence of more studies, preoperative chemotherapy may be considered in cases of very large tumors, grade 3 disease, an aggressive clinical course, or complete resection with high risk of morbidity. Usually, anthracycline is associated with ifosfamide. Clinical studies have observed an improvement in overall survival with an odds ratio of 0.56 and a reduction in absolute risk by 12%. However, it is only used in patients with a very high risk of recurrence. Patients with unresectable, locally advanced, or metastatic tumors can be treated with systemic therapy (Figure 7). The most commonly used is doxorubicin 75 mg/m^2^/cycle. A second line of treatment includes gemcitabine with docetaxel, trabectedin, or eribulin. Gemcitabine with docetaxel most often responds within 24 weeks. The best response is stable disease. When using trabectedin and eribulin, the toxicity profile must be taken into account. Other new therapies considered consist of targeting the pathogenic chain. CDK4/6 inhibitors can lead to cell growth arrest by inducing senescence and augmenting the tumor immune cell infiltrate. Palbociclib or abemaciclib can be used in this regard. In clinical studies, a median PFS of 17.9 weeks and 30 weeks was observed for the first and second agents, respectively [95,96,97]. MDM2 inhibitors such as milademethane are being considered. However, an increase in p53 levels was observed, which further induces MDM2 expression, providing a mechanism of chemoresistance [92]. Anti-PD therapy (pembrolizumab) requires continued investigation because initial results were not satisfactory [98]. Nivolumab also provided modest results [99]. Selinexor, an exportin inhibitor, showed promising results in terms of progression-free survival [100].

**Figure 8:**
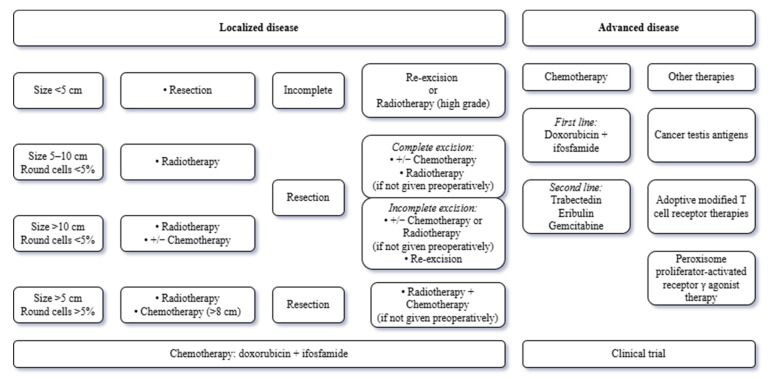
Schematic of therapeutic options for myxoid liposarcoma.

ML is the most chemoresponsive liposarcoma (Figure 8). However, treatment begins with surgical excision if its dimensions are less than 5 cm. If the excision is incomplete, it is completed with either re-excision or radiotherapy if the grade is high. In the case of tumors between 5 and 10 cm, with a histological component of round cells below 5%, treatment begins with radiotherapy and then surgical excision. After complete excision, chemotherapy can be associated, especially if it was not administered preoperatively or is of high grade. If the excision is incomplete, either re-excision or chemotherapy is performed. In liposarcomas with dimensions over 10 cm and a round cell component below 5%, radiotherapy and chemotherapy can be administered preoperatively. Depending on the excision, the procedure can be the same as for tumors under 10 cm. In liposarcomas with a round cell component of over 5%, preoperative radiotherapy (complete excision) is performed, which can also be associated with preoperative chemotherapy (if the size is over 8 cm). Preoperative chemotherapy is represented by doxorubicin with ifosfamide. In metastatic cases, the first line of chemotherapy provides anthracycline-based therapy (doxorubicin and ifosfamide). If there is a contraindication to anthracycline therapy, trabectedin can be administered. Secondary therapy provides trabectedin, if it has not been administered previously. Other therapies are represented by eribulin and gemcitabine. When available, patients should be referred to clinical trials. Other innovative therapies consist of the administration of cancer testis antigens (NY-ESO-1 and MAGE4) and adoptive modified T cell receptor (TCR) therapies. These have succeeded in reducing the disease control rate by half in clinical trials [101,102,103]. Peroxisome proliferator-activated receptor γ (PPARγ) agonist therapy, represented by pioglitazone associated with trabectedin, has shown a potentiation of the latter’s effect and is being investigated as a future therapeutic method [102,104].

PL treatment is represented by surgical excision and neoadjuvant and adjuvant radiotherapy. This method managed to reduce mortality by 63.7% [105,106]. Unfortunately, due to its aggressiveness, over 50% of patients develop metastases and have low overall survival. Thus, some studies recommend the use of neoadjuvant chemotherapy in eligible patients and those with tumors over 5 cm. The most used therapy remains the combination of doxorubicin and ifosfamide. In metastatic cases, therapeutic management provides the same management as in the case of DL or ML, but with moderate activity in these entities. The low incidence limits the inclusion of cases in clinical trials, resulting in a lack of information about the most effective treatment [99,101]. Treatment with aurora kinase inhibitors (AMG 900) reduced cell proliferation and survival, inducing apoptosis. Furthermore, combined treatment with doxorubicin enhanced its effect. When analyzing the kinome of cell lines after treatment with AMG 900, it was found that the inhibition of the MAPK pathway could be caused by the effects of the medication. Therefore, it could be an encouraging therapy [107].

Given the rarity of cases, there are no consensus recommendations regarding local and systemic therapy standards for MPL. Surgical excision is applied in combination with other therapeutic strategies. Tumors may respond to doxorubicin and ifosfamide, and eribulin and trabectedin are solutions for advanced stages. Most studies have failed to find targeted therapies against the *TP53* and *RB* pathways. Therefore, more studies are needed to develop new therapies for this entity [99,108].

### 4.4. Prognostic Factors

The overall prognosis of liposarcomas is influenced by multiple factors, such as sex, age, race, histological grade, staging, and tumor size. Relapse-free survival is determined primarily by histological subtype, tumor location, and surgical resection margins [109,110,111]. In order for disease-free survival to be possible and the risk of metastasis to be reduced, the tumor must be well differentiated, small in size, located in the extremities, and the resection margin must be negative [112,113]. Patients with high-grade liposarcomas (PL and DL) have a worse prognosis than those with better-differentiated forms (WDL and ML) (Table 4) [111].

ALT/WDL has the best prognosis of the five types of liposarcomas, with tumor location being the main predictor of recurrence [6,109]. The 5-year survival rate is 82%, and the 10-year survival rate is 68%. Soft tissue location in the trunk area has a poor prognosis, while limb location has a 0% mortality rate with complete excision [90,109]. It also does not show a significant correlation with tumor size, although large lesions are associated with a poor outcome. Radiotherapy provides little benefit, and these tumors are resistant to chemotherapy [6,109]. Regarding the risk of recurrence, up to 40% of ALT/WDL patients are at risk of recurrence if the tumor is located in the retroperitoneum, while limb location has a risk of recurrence of approximately 2% [114]. Well-differentiated liposarcomas may undergo a process of dedifferentiation, leading to increased tumor aggressiveness and a higher risk of recurrence and metastasis, especially if the lesion is located retroperitoneally [6,50].

DL presents a worse prognosis [7,109,115]. However, surgery performed with negative excision margins positively influences the prognosis [6,50]. The risk of recurrence and metastasis is on average 20%, being mainly influenced by the location of the lesion [114]. For tumors located in the extremities, the risk of recurrence is 20–30%, reaching up to 40% for retroperitoneal localization. Up to 30% of patients present metastases, most frequently in the lungs, followed by bone, liver, and retroperitoneal localizations. The median survival time of patients with dedifferentiated liposarcomas is 4.5 years, with a 5-year survival rate of 48% and a 10-year survival rate of 31% [50,109].

ML has an intermediate prognosis, depending on the percentage of round cells [50,116]. Thus, for tumors with less than 5% round cells, the risk of metastasis is 10–15%, with a 5-year survival rate of up to 80%. In the case of tumors with a percentage of round cells between 5 and 25%, the risk of metastasis reaches 40%, and 5-year survival drops to 70%. High-grade tumors (with more than 25% round cells) are the most aggressive, with a risk of metastasis of 60% and a 5-year survival of up to 50% [9,50]. The most common location of metastases is in the lung, followed by the liver, bone, and retroperitoneum [9,50,113]. Tumor size and advanced age significantly influence the prognosis. Thus, lesions larger than 10 cm are associated with a poor prognosis [109,116].

PL has a 5-year survival rate of 52% and a 10-year survival rate of 38%, with a median survival time of approximately 17 years. Radiotherapy, through its protective effect and complete excision of the lesion, provides a favorable outcome [6,109]. Negative prognostic factors are tumor size greater than 10 cm, high mitotic activity (greater than 20 mitoses/10 HPF), extensive necrosis (in greater than 50% of the tumor volume), and location. Location in the trunk or retroperitoneum is a negative prognostic factor. In general, the prognosis of this subtype of liposarcoma is unfavorable, as it has a high rate of local recurrence and metastasis [6,50,115]. Approximately 30–50% of these metastasize; the most common location is the lung [50].

MPL is a very rare tumor, with extreme aggressiveness, presenting a high recurrence rate and a very high risk of metastasis. The prognosis of this tumor type is more unfavorable compared to pleomorphic and myxoid liposarcomas. The median survival time for MPL is only 22.6 months, compared to PL (75.9 months) and ML (218.3 months) [16,43].

### 4.5. Limitations

This scoping review has some inherent methodological limitations, and these should be considered when evaluating the findings. The literature search was thorough and performed across three major databases. The search was restricted to publications from recent years (2016–2026) and exclusively to research published in English. This method may have resulted in the exclusion of relevant historical data or unique regional findings published in other languages. We employed a rigorous approach; however, the study protocol was not prospectively registered in a public registry (e.g., PROSPERO), which could be considered a perceived limitation on ideal openness. We assessed the quality of the evidence, which added methodological strength. Due to the great heterogeneity of the study designs included (from primary investigations to systematic reviews), we were not able to do a quantitative synthesis or meta-analysis. Thus, the results could only be pooled by use of a descriptive thematic analysis, limiting our capacity to make definite, statistically pooled clinical recommendations for the care of liposarcoma.

## 5. Conclusions

Liposarcomas present different subtypes with different biological and clinical behaviors. The careful integration of history and clinical manifestations, imaging findings, and histopathology, completed by immunohistochemistry and molecular testing, is an essential requirement for accurate diagnosis. It is important to remember that none of these examinations can provide the diagnosis alone, considering the evaluation of the differential diagnosis. Each tool contributes equally to the patient’s chance. Clinical and imaging findings guide decision-making, therapeutic plans, and prognosis. Histopathology, along with immunohistochemistry and molecular biology, plays a crucial role in providing an accurate diagnosis and informing the therapeutic regimen.

However, we are in an early stage of liposarcoma management. There must be more studies to support discoveries and their applicability, such as the integration of machine learning tools in increasing the accuracy of imaging examinations and the discovery of new modifications of pathogenic chains to discover new immunohistochemical and molecular markers. All of these are necessary for the individualization of treatment and increasing its accuracy. Continuous collaborations between specialties at the international level are essential for increasing research efforts, rapidly establishing new systemic therapies or improving existing ones, and enhancing research and translational medicine. All of this has as its beneficiary the patient whose disease-free survival and overall survival are as high as possible, with the reduction in therapeutic side effects and the improvement in quality of life.

## Figures and Tables

**Table 4:** The main prognostic aspects of liposarcomas.

Tumor	Prognosis	Predictor of Recurrence	Survival	Metastasis
Atypical lipomatous tumor/well-differentiated liposarcoma [6,90,109,114]	Good	Tumor location (retroperitoneal 40%, extremities 2%)	82% at 5 years 68% at 10 years	-
Dedifferentiated liposarcoma [6,7,50,109,114,115]	Poor	Incomplete excision Tumor location (extremities 20–30%; retroperitoneal 40%)	48% at 5 years 31% at 10 years	Up to 30% of cases (lung, bones, liver)
Myxoid liposarcoma [9,50,109,113,116]	Intermediate	Tumor size (>10 cm) Old age	<5% round cells: 80% at 5 years 5–25% round cells: 70% at 5 years >25% round cells: 50% at 5 years	<5% round cells: 10–15% risk 5–25% round cells: 40% risk >25% round cells: 60% risk
Pleomorphic liposarcoma [6,50,109,115]	Poor	Tumor size (>10 cm) Mitotic activity (>20 mitoses/10 HPF) Tumor necrosis (>50%)	52% at 5 years 38% at 10 years	Approximately 30–50% (lung)
Myxoid pleomorphic liposarcoma [16,43]	Very poor	Very high rate	Median 22.6 months	Risk of over 40%

## Data Availability

No new data were created or analyzed in this study. Data sharing is not applicable to this article.

## References

[1] WHO Classification of Tumours Editorial Board (2020). Soft Tissue and Bone Tumours.

[2] Amer K.M., Congiusta D.V., Thomson J.E., Elsamna S., Chaudhry I., Bozzo A., Amer R., Siracuse B., Ghert M., Beebe K.S. (2020). Epidemiology and survival of liposarcoma and its subtypes: A dual database analysis. J. Clin. Orthop. Trauma.

[3] Dwianingsih E.K., Bawono R.G., Saputri A., Malueka R.G., Putro Y.A.P., Anwar S.L., Widodo I. (2025). Histomorphological and molecular characteristics of liposarcoma (Review). Oncol. Lett..

[4] Wu J., Zhang Z., Song F., Chen X., Huang S. (2025). Developing a diagnostic model to differentiate the well-differentiated lipomatous tumors based on clinicopathological characteristics. Sci. Rep..

[5] Bock S., Hoffmann D.G., Jiang Y., Chen H., Il’yasova D. (2020). Increasing Incidence of Liposarcoma: A Population-Based Study of National Surveillance Databases, 2001–2016. Int. J. Environ. Res. Public Health.

[6] Jonczak E., Grossman J., Alessandrino F., Seldon Taswell C., Velez-Torres J.M., Trent J. (2024). Liposarcoma: A Journey into a Rare Tumor’s Epidemiology, Diagnosis, Pathophysiology, and Limitations of Current Therapies. Cancers.

[7] Thway K. (2019). Well-differentiated liposarcoma and dedifferentiated liposarcoma: An updated review. Semin. Diagn. Pathol..

[8] Obeidat A. (2026). Predictors of survival in dedifferentiated liposarcoma: A population-based analysis of the SEER database. Medicine.

[9] Muratori F., Bettini L., Frenos F., Mondanelli N., Greto D., Livi L., Franchi A., Roselli G., Scorianz M., Capanna R. (2018). Myxoid Liposarcoma: Prognostic Factors and Metastatic Pattern in a Series of 148 Patients Treated at a Single Institution. Int. J. Surg. Oncol..

[10] Papalia G.F., De Marco G., Luciano C., Sisca L., Farsetti P., Vincenzi B., Papalia R. (2025). Evaluation of Prognostic Factors in Myxoid Liposarcoma Treated with Combined Neoadjuvant Radiotherapy and Surgical Excision: Systematic Review. Diseases.

[11] Coombs R.A., Jebastin Thangaiah J., Siontis B.L., Robinson S.I., Okuno S.H., Houdek M.T., Xu-Welliver M., Ho T.P. (2024). Tolerability and Outcomes for Treatment of Older Myxoid Liposarcoma Population. Cancers.

[12] Nasir I., Yeap B.T., Koo T.H., Paul A.G., Zakaria M.H. (2025). Unveiling the enigma of myxoid liposarcoma: Diagnostic challenges and multidisciplinary triumphs in limb-salvage surgery. Radiol. Case Rep..

[13] Wan L., Tu C., Qi L., Li Z. (2021). Survivorship and prognostic factors for pleomorphic liposarcoma: A population-based study. J. Orthop. Surg. Res..

[14] Shimomura S., Shirai T., Terauchi R., Mizoshiri N., Mori Y., Inuma K., Tsuchida S., Morimura R., Ikoma H. (2023). Pleomorphic liposarcoma of the extremity with solitary huge liver metastasis at initial diagnosis treated with conversion surgery combined with adjuvant chemotherapy: A case report. J. Med. Case Rep..

[15] Creytens D., Folpe A.L., Koelsche C., Mentzel T., Ferdinande L., van Gorp J.M., Van der Linden M., Raman L., Menten B., Fritchie K. (2021). Myxoid pleomorphic liposarcoma—A clinicopathologic, immunohistochemical, molecular genetic and epigenetic study of 12 cases, suggesting a possible relationship with conventional pleomorphic liposarcoma. Mod. Pathol..

[16] Nishio J., Nakayama S., Aoki M. (2026). Myxoid Pleomorphic Liposarcoma: A Review and Update. Cancer Genom. Proteom..

[17] Arksey H., O’Malley L. (2005). Scoping studies: Towards a methodological framework. Int. J. Soc. Res. Methodol..

[18] Peters M.D.J., Godfrey C.M., McInerney P., Munn Z., Tricco A.C., Khalil H., Aromataris E., Munn Z. (2020). Chapter 11: Scoping Reviews (2020 version). JBI Manual for Evidence Synthesis.

[19] Tricco A.C., Lillie E., Zarin W., O’Brien K.K., Colquhoun H., Levac D., Moher D., Peters M.D.J., Horsley T., Weeks L. (2018). PRISMA Extension for Scoping Reviews (PRISMA-ScR): Checklist and Explanation. Ann. Intern. Med..

[20] Johnson C.N., Ha A.S., Chen E., Davidson D. (2018). Lipomatous Soft-tissue Tumors. J. Am. Acad. Orthop. Surg..

[21] Keung E.Z., Ikoma N., Benjamin R., Wang W.L., Lazar A.J., Feig B.W. (2018). The clinical behavior of well differentiated liposarcoma can be extremely variable: A retrospective cohort study at a major sarcoma center. J. Surg. Oncol..

[22] Natella R., Varriano G., Brunese M.C., Zappia M., Bruno M., Gallo M., Fazioli F., Simonetti I., Granata V., Brunese L. (2023). Increasing differential diagnosis between lipoma and liposarcoma through radiomics: A narrative review. Explor. Target. Antitumor Ther..

[23] Muhib M., Abidi S.L.F., Ahmed U., Afzal A., Farooqui A., Khalid Jamil O.B., Ahmed S., Agha H. (2024). Use of radiologic imaging to differentiate lipoma from atypical lipomatous tumor/well-differentiated liposarcoma: Systematic review. SAGE Open Med..

[24] Casier J., Timmermans I., Laenen A., Hompes D., Douchy T., Sciot R., Christiaens M., Wafa H., Schöffski P. (2025). Clinical course and prognostic factors of patients with dedifferentiated liposarcoma: A retrospective analysis. BMC Cancer.

[25] Zhang T., Liu B. (2024). MRI Differential Diagnosis and Guidance for Puncture Biopsy of Musculoskeletal Dedifferentiated Liposarcoma and Well Differentiated Liposarcoma. Cancer. Manag. Res..

[26] Yang T., Chen R.Y., Ding Y.F., Wu J.Y., Li Y., Qiang J.W. (2025). CT-based radiomics nomogram for differentiating dedifferentiated liposarcoma from well-differentiated liposarcoma. Front. Oncol..

[27] Shimamori N., Kishino T., Morii T., Okabe N., Motohashi M., Matsushima S., Yamasaki S., Ohtsuka K., Shibahara J., Ichimura S. (2019). Sonographic Appearances of Liposarcoma: Correlations with Pathologic Subtypes. Ultrasound Med. Biol..

[28] Parkes A., Urquiola E., Bhosale P., Lin H., Watson K., Wang W.L., Feig B., Torres K., Roland C.L., Conley A.P. (2020). PET/CT Imaging as a Diagnostic Tool in Distinguishing Well-Differentiated versus Dedifferentiated Liposarcoma. Sarcoma.

[29] Scalas G., Parmeggiani A., Martella C., Tuzzato G., Bianchi G., Facchini G., Clinca R., Spinnato P. (2021). Magnetic resonance imaging of soft tissue sarcoma: Features related to prognosis. Eur. J. Orthop. Surg. Traumatol..

[30] Kawaguchi M., Kato H., Kobayashi K., Fujishiro S., Furui T., Miyazaki T., Goshima S., Matsuo M. (2022). MRI findings to differentiate musculoskeletal dedifferentiated liposarcoma from atypical lipomatous tumor. Radiol. Med..

[31] Abaricia S., Hirbe A.C. (2018). Diagnosis and Treatment of Myxoid Liposarcomas: Histology Matters. Curr. Treat. Options Oncol..

[32] de Boer H.C., Musson R. (2023). Imaging features of myxoid soft-tissue tumours. Clin. Radiol..

[33] Correa N., Kumar M., Gonzalez J., Martinez L., Alexander A., Manzur K., Bermudez F. (2026). Rare Myxoid Liposarcoma of the Thigh: A Case Report. Dermato.

[34] Kawaguchi M., Kato H., Kobayashi K., Miyazaki T., Nagano A., Noda Y., Hyodo F., Matsuo M. (2025). Features of MR Imaging that Differentiate between Immunohistochemically Diagnosed Dedifferentiated Liposarcoma and Myxoid Liposarcoma. Magn. Reson. Med. Sci..

[35] Saifuddin A., Andrei V., Rajakulasingam R., Oliveira I., Seddon B. (2021). Magnetic resonance imaging of trunk and extremity myxoid liposarcoma: Diagnosis, staging, and response to treatment. Skelet. Radiol..

[36] Gimber L.H., Montgomery E.A., Morris C.D., Krupinski E.A., Fayad L.M. (2017). MRI characteristics associated with high-grade myxoid liposarcoma. Clin. Radiol..

[37] Wang L., Luo R., Xiong Z., Xu J., Fang D. (2018). Pleomorphic liposarcoma: An analysis of 6 case reports and literature review. Medicine.

[38] Lee A.T.J., Thway K., Huang P.H., Jones R.L. (2018). Clinical and Molecular Spectrum of Liposarcoma. J. Clin. Oncol..

[39] Anderson W.J., Jo V.Y. (2019). Pleomorphic liposarcoma: Updates and current differential diagnosis. Semin. Diagn. Pathol..

[40] Jo S.J., Jung H.K., Nam K.H. (2020). Recurrent Primary Pleomorphic Liposarcoma of the Breast: A Case Report with Imaging Findings. J. Breast Cancer.

[41] Burt A.M., Huang B.K. (2017). Imaging review of lipomatous musculoskeletal lesions. SICOT J..

[42] Dermawan J.K., Hwang S., Wexler L., Tap W.D., Singer S., Vanderbilt C.M., Antonescu C.R. (2022). Myxoid pleomorphic liposarcoma is distinguished from other liposarcomas by widespread loss of heterozygosity and significantly worse overall survival: A genomic and clinicopathologic study. Mod. Pathol..

[43] Fadaei S., Cordier F., Ferdinande L., Van Dorpe J., Creytens D. (2024). Myxoid pleomorphic liposarcoma. Histol. Histopathol..

[44] AlObaid B., Alzahrani N.A., Shokor N., Alshammari K. (2022). Myxoid pleomorphic liposarcoma of the falciform ligament: A rare case report. J. Surg. Case Rep..

[45] Chandrasekaran Y., Amitkumar K., Elamaran A., Sudalaimuthu M., Kumaran S. (2024). Myxoid pleomorphic liposarcoma of the spermatic cord: A rare entity at a rare site. Cureus.

[46] Shen Y., Zhao L., Li A., Peng Q., Liu Q., Wang L., Liu Z. (2024). Rare myxoid pleomorphic liposarcoma: A case report and literature review. J. Clin. Pathol..

[47] Dermawan J.K. (2024). Myxoid pleomorphic liposarcoma. Surg. Pathol. Clin..

[48] Al Kindi A.H., Al Kindi F.A., Al Riyami M., Khalil E. (2023). Giant mediastinal myxoid pleomorphic liposarcoma. Sultan Qaboos Univ. Med. J..

[49] Choi J.H., Lee S.H., Kim K.S., Choi Y.D., Hwang J.H., Lee S.Y. (2022). Myxoid pleomorphic liposarcoma in the teres minor muscle: A case report. Medicine.

[50] Menon G., Kaur A. (2026). Liposarcoma. StatPearls.

[51] Mashima E., Sawada Y., Nakamura M. (2021). Recent Advancement in Atypical Lipomatous Tumor Research. Int. J. Mol. Sci..

[52] Lu J., Wood D., Ingley E., Koks S., Wong D. (2021). Update on genomic and molecular landscapes of well-differentiated liposarcoma and dedifferentiated liposarcoma. Mol. Biol. Rep..

[53] Choi J.H., Ro J.Y. (2020). Retroperitoneal Sarcomas: An Update on the Diagnostic Pathology Approach. Diagnostics.

[54] Kammerer-Jacquet S.F., Thierry S., Cabillic F., Lannes M., Burtin F., Henno S., Dugay F., Bouzillé G., Rioux-Leclercq N., Belaud-Rotureau M.-A. (2017). Differential diagnosis of atypical lipomatous tumor/well-differentiated liposarcoma and dedifferentiated liposarcoma: Utility of p16 in combination with MDM2 and CDK4 immunohistochemistry. Hum. Pathol..

[55] Soraya F., Sandhika W. (2024). Immunohistochemistry assay for differentiating liposarcoma and its mimickers. Int. J. Sci. Adv..

[56] Khan W.F., Rathore Y.S., Aduri R.S., Mridha A.R. (2019). Lipoleiomyosarcoma of spermatic cord: An unusual presentation. BMJ Case Rep..

[57] Suster D.I., Suster S. (2020). Liposarcomas of the mediastinum. Mediastinum.

[58] Kukull B.J., Khalighi M.A., Gundle K.R., Hansford B.G., Corless C.L., Davis J.L. (2020). Low-grade Osteosarcomatous Dedifferentiation of an Atypical Lipomatous Tumor in a Pediatric Patient. Pediatr. Dev. Pathol..

[59] Macagno N., Fuentes S., de Pinieux G., Maues de Paula A., Salas S., Mattéi J.C., Dupuis C., Appay R., Aurias A., Dufour H. (2017). Paravertebral Well-Differentiated Liposarcoma with Low-Grade Osteosarcomatous Component: Case Report with 11-Year Follow-Up, Radiological, Pathological, and Genetic Data, and Literature Review. Case Rep. Pathol..

[60] Kilpatrick S.E. (2024). Atypical lipomatous tumor/well differentiated liposarcoma and related mimics with updates. When is molecular testing most cost-effective, necessary, and indicated?. Hum. Pathol..

[61] Nishio J., Nakayama S., Nabeshima K., Yamamoto T. (2021). Biology and Management of Dedifferentiated Liposarcoma: State of the Art and Perspectives. J. Clin. Med..

[62] Hirata M., Asano N., Katayama K., Yoshida A., Tsuda Y., Sekimizu M., Mitani S., Kobayashi E., Komiyama M., Fujimoto H. (2019). Integrated exome and RNA sequencing of dedifferentiated liposarcoma. Nat. Commun..

[63] Kurzawa P., Mullen J.T., Chen Y.L., Johnstone S.E., Deshpande V., Chebib I., Nielsen G.P. (2020). Prognostic Value of Myogenic differentiation in dedifferentiated liposarcoma. Am. J. Surg. Pathol..

[64] Wang G.Y., Lucas D.R. (2018). Dedifferentiated Liposarcoma with Myofibroblastic Differentiation. Arch. Pathol. Lab. Med..

[65] Dehner C.A., Hagemann I.S., Chrisinger J.S.A. (2021). Retroperitoneal Dedifferentiated Liposarcoma. Am. J. Clin. Pathol..

[66] Bourgeau M., Gandhi J.S., Deeb K.K., Bahrami A. (2024). Superficial dedifferentiated liposarcoma: A clinicopathologic study. Hum. Pathol..

[67] Usman Tariq M., Kayani N., Moatter T., Din N.U. (2020). Dedifferentiated Liposarcoma with Meningothelial-Like Whorls: Five Additional Cases and Review of the Literature. Int. J. Surg. Pathol..

[68] Song M.J., Cho K.J., Lee J.S., Song J.S. (2017). Application of MDM2 Fluorescence In Situ Hybridization and Immunohistochemistry in Distinguishing Dedifferentiated Liposarcoma from Other High-grade Sarcomas. Appl. Immunohistochem. Mol. Morphol..

[69] Anderson W.J., Jo V.Y. (2021). Diagnostic Immunohistochemistry of Soft Tissue and Bone Tumors: An Update on Biomarkers That Correlate with Molecular Alterations. Diagnostics.

[70] Yang L., Chen S., Luo P., Yan W., Wang C. (2020). Liposarcoma: Advances in Cellular and Molecular Genetics Alterations and Corresponding Clinical Treatment. J. Cancer.

[71] Åman P., Dolatabadi S., Svec D., Jonasson E., Safavi S., Andersson D., Grundevik P., Thomsen C., Ståhlberg A. (2016). Regulatory mechanisms, expression levels and proliferation effects of the FUS-DDIT3 fusion oncogene in liposarcoma. J. Pathol..

[72] Trautmann M., Menzel J., Bertling C., Cyra M., Isfort I., Steinestel K., Elges S., Grünewald I., Altvater B., Rossig C. (2017). FUS–DDIT3 fusion protein-driven IGF-IR signaling is a therapeutic target in myxoid liposarcoma. Clin. Cancer Res..

[73] Nezu Y., Hagiwara K., Yamamoto Y., Fujiwara T., Matsuo K., Yoshida A., Kawai A., Saito T., Ochiya T. (2016). miR-135b, a key regulator of malignancy, is linked to poor prognosis in human myxoid liposarcoma. Oncogene.

[74] Qi Y., Hu Y., Yang H., Zhuang R., Hou Y., Tong H., Feng Y., Huang Y., Jiang Q., Ji Q. (2017). Establishing a patient-derived xenograft model of human myxoid and round-cell liposarcoma. Oncotarget.

[75] Creytens D. (2019). A contemporary review of myxoid adipocytic tumors. Semin. Diagn. Pathol..

[76] Qu G., Zhang C., Tian Z., Yao W. (2024). Diagnosis and Treatment of Myxoid Liposarcoma. Curr. Treat. Options Oncol..

[77] Scapa J.V., Cloutier J.M., Raghavan S.S., Peters-Schulze G., Varma S., Charville G.W. (2021). DDIT3 Immunohistochemistry Is a Useful Tool for the Diagnosis of Myxoid Liposarcoma. Am. J. Surg. Pathol..

[78] Kojima N., Kubo T., Mori T., Satomi K., Matsushita Y., Iwata S., Yatabe Y., Ichimura K., Kawai A., Ichikawa H. (2024). Myxoid liposarcoma with nuclear pleomorphism: A clinicopathological and molecular study. Virchows Arch..

[79] Baranov E., Black M.A., Fletcher C.D.M., Charville G.W., Hornick J.L. (2021). Nuclear expression of DDIT3 distinguishes high-grade myxoid liposarcoma from other round cell sarcomas. Mod. Pathol..

[80] Tyler R., Wanigasooriya K., Taniere P., Almond M., Ford S., Desai A., Beggs A. (2020). A review of retroperitoneal liposarcoma genomics. Cancer Treat. Rev..

[81] M.S A., K C., Bhargavan R.V., Somanathan T., Subhadradevi L. (2024). An overview on liposarcoma subtypes: Genetic alterations and recent advances in therapeutic strategies. J. Mol. Histol..

[82] Hornick J.L. (2018). Subclassification of pleomorphic sarcomas: How and why should we care?. Ann. Diagn. Pathol..

[83] Ciongariu A.M., Țăpoi D.A., Dumitru A.V., Bejenariu A., Marin A., Costache M. (2024). Pleomorphic Liposarcoma Unraveled: Investigating Histopathological and Immunohistochemical Markers for Tailored Diagnosis and Therapeutic Innovations. Medicina.

[84] Carvalho S.D., Pissaloux D., Crombé A., Coindre J.M., Le Loarer F. (2019). Pleomorphic Sarcomas: The State of the Art. Surg. Pathol. Clin..

[85] Abe M., Hoshi N., Hoshi S., Hirabayashi K., Kikuta K., Hirozane T., Nakagawa R., Mizuno T., Nakamura H., Inoue K. (2023). A Case of GATA3 Positive Pleomorphic Liposarcoma, Epithelioid Variant: A Diagnostic Pitfall. Case Rep. Pathol..

[86] Sbaraglia M., Bellan E., Dei Tos A.P. (2021). The 2020 WHO Classification of Soft Tissue Tumours: News and perspectives. Pathologica.

[87] Refai F. (2019). The histopathological grading of soft tissue sarcomas: A review. Saudi J. Pathol. Microbiol..

[88] Liu H., Wang X., Wang X., Qiu F., Zhou B. (2025). Challenges and hope: Latest research trends in the clinical treatment and prognosis of liposarcoma. Front. Pharmacol..

[89] Muratori F., Frenos F., Bettini L., Matera D., Mondanelli N., Scorianz M., Cuomo P., Scoccianti G., Beltrami G., Greto D. (2018). Liposarcoma: Clinico-pathological analysis, prognostic factors and survival in a series of 307 patients treated at a single institution. J. Orthop. Sci..

[90] Schöffski P. (2022). Established and Experimental Systemic Treatment Options for Advanced Liposarcoma. Oncol. Res. Treat..

[91] Mansfield S.A., Pollock R.E., Grignol V.P. (2018). Surgery for Abdominal Well-Differentiated Liposarcoma. Curr. Treat. Options Oncol..

[92] Haddox C.L., Hornick J.L., Roland C.L., Baldini E.H., Keedy V.L., Riedel R.F. (2024). Diagnosis and management of dedifferentiated liposarcoma: A multidisciplinary position statement. Cancer Treat. Rev..

[93] ASTRO (2021). Soft Tissue Sarcoma in Adults: Executive Summary of an ASTRO Clinical Practice Guideline. Pract. Radiat. Oncol..

[94] Gronchi A., Miah A.B., Dei Tos A., Abecassis N., Bajpai J., Bauer S., Biagini R., Bielack S., Blay J.Y., Bolle S. (2021). Soft tissue and visceral sarcomas: ESMO-EURACAN-GENTURIS Clinical Practice Guidelines for diagnosis, treatment and follow-up. Ann. Oncol..

[95] Gahvari Z., Parkes A. (2020). Dedifferentiated Liposarcoma: Systemic Therapy Options. Curr. Treat. Options Oncol..

[96] Dickson M.A., Schwartz G.K., Keohan M.L., D’angelo S.P., Gounder M.M., Chi P., Antonescu C.R., Landa J., Qin L.-X., Crago A.M. (2016). Progression-free survival among patients with well-differentiated or dedifferentiated liposarcoma treated with CDK4 inhibitor Palbociclib: A phase 2 clinical trial. JAMA Oncol..

[97] Dickson M.A., Koff A., D’ANgelo S.P., Gounder M.M., Keohan M.L., Kelly C.M., Chi P., Antonescu C.R., Landa J., Qin L.-X. (2019). Phase 2 study of the CDK4 inhibitor abemaciclib in dedifferentiated liposarcoma. J. Clin. Oncol..

[98] Burgess M.A., Bolejack V., Schuetze S., Van Tine B.A., Attia S., Riedel R.F., Hu J.S., Davis L.E., Okuno S.H., Priebat D.A. (2019). Clinical activity of pembrolizumab (P) in undifferentiated pleomorphic sarcoma (UPS) and dedifferentiated/pleomorphic liposarcoma (LPS): Final results of SARC028 expansion cohorts. J. Clin. Oncol..

[99] Haddox C.L., Riedel R.F. (2021). Recent advances in the understanding and management of liposarcoma. Fac. Rev..

[100] Gounder M., Razak A.R.A., Gilligan A.M., Leong H., Ma X., Somaiah N., Chawla S.P., Martin-Broto J., Grignani G., Schuetze S.M. (2021). Health-related quality of life and pain with selinexor in patients with advanced dedifferentiated liposarcoma. Future Oncol..

[101] Crago A.M., Dickson M.A. (2016). Liposarcoma: Multimodality Management and Future Targeted Therapies. Surg. Oncol. Clin. N. Am..

[102] Nassif E.F., Keung E.Z., Thirasastr P., Somaiah N. (2023). Myxoid Liposarcomas: Systemic Treatment Options. Curr. Treat. Options Oncol..

[103] D’Angelo S.P., Van Tine B.A., Attia S., Blay J.-Y., Strauss S.J., Valverde Morales C.M., Abdul Razak A.R., Van Winkle E., Trivedi T., Biswas S. (2021). SPEARHEAD-1: A phase 2 trial of afamitresgene autoleucel (formerly ADP-A2M4) in patients with advanced synovial sarcoma or myxoid/round cell liposarcoma. J. Clin. Oncol..

[104] Frapolli R., Bello E., Ponzo M., Craparotta I., Mannarino L., Ballabio S., Marchini S., Carrassa L., Ubezio P., Porcu L. (2019). Combination of PPARγ agonist pioglitazone and trabectedin induce adipocyte differentiation to overcome trabectedin resistance in myxoid liposarcomas. Clin. Cancer Res..

[105] Assi T., Ngo C., Faron M., Verret B., Lévy A., Honoré C., Hénon C., Le Péchoux C., Bahleda R., Le Cesne A. (2023). Systemic Therapy in Advanced Pleomorphic Liposarcoma: A Comprehensive Review. Curr. Treat. Options Oncol..

[106] Lee H.G., Aurit S., Silberstein P., Gootee J. (2020). Primary anatomical site as a prognostic factor for pleomorphic liposarcoma. J. Cancer Res. Clin. Oncol..

[107] Casadei L., de Faria F.C.C., Lopez-Aguiar A., Pollock R.E., Grignol V. (2022). Targetable Pathways in the Treatment of Retroperitoneal Liposarcoma. Cancers.

[108] Lesovaya E.A., Fetisov T.I., Bokhyan B.Y., Maksimova V.P., Kulikov E.P., Belitsky G.A., Kirsanov K.I., Yakubovskaya M.G. (2024). Genetic, Epigenetic and Transcriptome Alterations in Liposarcoma for Target Therapy Selection. Cancers.

[109] Zhao J., Du W., Tao X., Li A., Li Y., Zhang S. (2025). Survival and prognostic factors among different types of liposarcomas based on SEER database. Sci. Rep..

[110] Sun P., Ma R., Liu G., Wang L., Chang H., Li Y. (2021). Pathological prognostic factors of retroperitoneal liposarcoma: Comprehensive clinicopathological analysis of 124 cases. Ann. Transl. Med..

[111] Oh Y.J., Yi S.Y., Kim K.H., Cho Y.J., Beum S.H., Lee Y.H., Suh J.-S., Hur H., Kim K.S., Kim S.H. (2016). Prognostic Model to Predict Survival Outcome for Curatively Resected Liposarcoma: A Multi-Institutional Experience. J. Cancer.

[112] Knebel C., Lenze U., Pohlig F., Lenze F., Harrasser N., Suren C., Breitenbach J., Rechl H., von Eisenhart-Rothe R., Mühlhofer H.M.L. (2017). Prognostic factors and outcome of Liposarcoma patients: A retrospective evaluation over 15 years. BMC Cancer.

[113] Lin F., Duan J., Lin Y., Wu H., Xu G., Guo X., Liu Z., Xu Y., Mao M., Wang X. (2020). Survival and risk factors in patients with liposarcoma with distant metastasis. Am. J. Transl. Res..

[114] Bill K.L., Casadei L., Prudner B.C., Iwenofu H., Strohecker A.M., Pollock R.E. (2016). Liposarcoma: Molecular targets and therapeutic implications. Cell. Mol. Life Sci..

[115] Ciongariu A.M., Țăpoi D.A., Dumitru A.V., Enache V., Marin A., Creangă C.A., Costache M. (2025). Enhancing Liposarcoma Prognosis-A New Predictive Scoring System Integrating Histopathological Insights. Cancer Manag. Res..

[116] Tfayli Y., Baydoun A., Naja A.S., Saghieh S. (2021). Management of myxoid liposarcoma of the extremity. Oncol. Lett..

